# Maturation of the [Ni–4Fe–4S] active site of carbon monoxide dehydrogenases

**DOI:** 10.1007/s00775-018-1541-0

**Published:** 2018-02-14

**Authors:** Mériem Merrouch, Martino Benvenuti, Marco Lorenzi, Christophe Léger, Vincent Fourmond, Sébastien Dementin

**Affiliations:** Aix-Marseille Université, CNRS, BIP UMR 7281, Institut de Microbiologie de la Méditerranée, 31 Chemin J. Aiguier, 13402 Marseille Cedex 20, France

**Keywords:** Carbon monoxide dehydrogenase, Active site, Iron–sulfur cluster, Maturation

## Abstract

Nickel-containing enzymes are diverse in terms of function and active site structure. In many cases, the biosynthesis of the active site depends on accessory proteins which transport and insert the Ni ion. We review and discuss the literature related to the maturation of carbon monoxide dehydrogenases (CODH) which bear a nickel-containing active site consisting of a [Ni–4Fe–4S] center called the C-cluster. The maturation of this center has been much less studied than that of other nickel-containing enzymes such as urease and NiFe hydrogenase. Several proteins present in certain CODH operons, including the nickel-binding proteins CooT and CooJ, still have unclear functions. We question the conception that the maturation of all CODH depends on the accessory protein CooC described as essential for nickel insertion into the active site. The available literature reveals biological variations in CODH active site biosynthesis.

## Introduction

Nine nickel-containing enzymes have been discovered and characterized so far, but there exists certainly others [1, 2]. Among them, seven consume or produce small molecules (hydrogenase, carbon monoxide dehydrogenase, superoxide dismutase, urease, acireductone dioxygenase, methyl-coM reductase and acetyl-CoA synthase). The other two (glyoxylase I and lactate racemase) are involved in lactate metabolism. The structures of their active sites are diverse in terms of nature and number of ligands to the Ni. In most cases, the Ni is coordinated by acidic residues (Cys, His, Glu, Asp, carbonylated Lys) or water molecules. The three exceptions are carbon monoxide dehydrogenase, in which Ni is also coordinated to inorganic sulfur in a [Ni–4Fe–4S] cluster, lactate racemase in which Ni is part of a non-protein cofactor (pincer) linked to a Lys of the protein backbone and methyl-coM reductase in which Ni is part of coenzyme F430. Depending on the enzyme, Ni participates in catalysis either by acting as a Lewis acid or by promoting redox chemistry.

Although the insertion of Ni seems spontaneous in glyoxylase I and acireductone dioxygenase, it requires dedicated biological machineries in the other cases. The present review focuses on the carbon monoxide dehydrogenases from anaerobic microorganisms (Ni-containing CODH) which catalyze the reversible oxidation of CO with high turnover frequencies [3–6]. These enzymes bear a nickel-containing active site, the so-called C-cluster, which consists of a [Ni–3Fe–4S] cubane connected to a unique iron site through a linking sulfide [7–9].

## Generalities

**Fig. 1:**
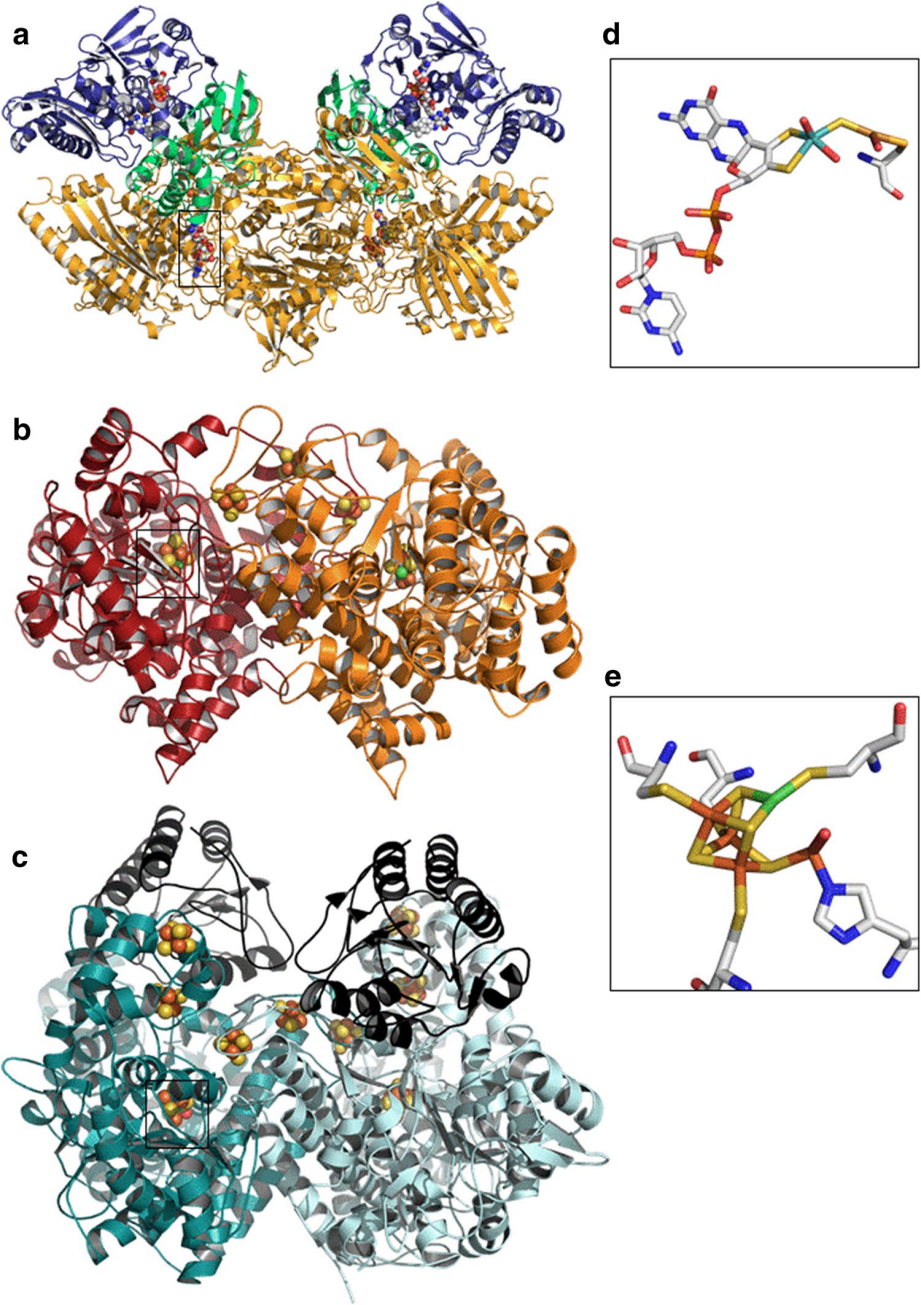
Structures of prototypical Ni-containing CODH and their active sites. **a** Structure of the MoCu-CODH from *Oligotropha carboxidovorans*, PDB: 1N63 [30]. The L-subunit (89 kDa) in yellow, contains the active site. The S-subunit (18 kDa) in green, contains the two [2Fe–2S] clusters and the M-subunit (30 kDa) binds a FAD cofactor. **b** Structure of the CODH-II from *Carboxydothermus hydrogenoformans* (Ch), PDB: 3B53 [31]. Each subunit (67 kDa) of this homodimer is colored in red or in orange. **c** Structure of the CODH from *Methanosarcina barkeri*, PDB: 3CF4 [32]. Subunit α (89 kDa) containing the nine iron–sulfur clusters is colored in blue and the subunit ε (20 kDa) is colored in black. Structures of the active sites of the MoCu–CODH from *Oligotropha carboxidovorans* (**d**) and of the CODH-II from Ch (**e**). The colors for the atoms in **d**, **e** are green for Ni, orange for Fe, yellow for S, red for O, blue for N, turquoise for Mo, light orange for Cu and white for C

Some microorganisms can grow in the presence of carbon monoxide, which they use as a source of carbon and/or energy [10, 11]. The oxidation of CO to CO_2_ by these microorganisms is catalyzed by carbon monoxide dehydrogenases (CODH). Nevertheless, CODH from aerobic and anaerobic bacteria are not phylogenetically related and have distinct structures and kinetic properties. Most aerobic CO-utilizing bacteria (carboxydotrophs) oxidize CO in their respiratory chain [12] using a variety of acceptors such as O_2_ (*Oligotropha carboxidovorans* [13]) or nitrate for dissimilatory nitrate reduction (*Burkholderia xenovorans LB400* [11]). Some photosynthetic bacteria, such as *Rhodopseudomonas gelatinosa*, can use CO as a carbon source by first converting it into CO_2_, which is then reduced into carbohydrate through the Calvin–Benson–Bassham cycle [14]. CODH from aerobic bacteria are heterotrimeric enzymes and belong to the xanthine oxidase family; their active site a binuclear cluster of Mo and Cu (MoCu–CODH) (Fig. 1a, d). These MoCu–CODH only catalyze the oxidation of CO (not the reduction of CO_2_) with a turnover frequency of up to 100 s^−1^ [13].

Here we focus on the CODH from anaerobic microorganisms (Ni-containing CODH), which we will abbreviate CODH in the following text for clarity. These enzymes contain a unique nickel-containing active site [Ni–4Fe–4S], called the C-cluster. This cluster consists of a [Ni–3Fe–4S] cubane connected to a unique iron site through a linking sulfide (Fig. 1e) [7–9]. CODH catalyze the oxidation of CO with turnover frequencies ranging from hundreds to tens of thousands turnovers per second [3–6]. CODH-I from *Carboxydothermus hydrogenoformans* (Ch) is the most active, with a turnover rate of 39,000 s^−1^ at 70 °C, pH 8 [3]. Contrary to Mo–CODH, CODH also catalyze the reverse reaction (the reduction of CO_2_ into CO). These enzymes are divided in four classes depending on their structures and functions [15]. CODH of classes I, II and III are bifunctional, they form a complex with acetyl-CoA synthase (ACS/CODH), while class IV CODH are monofunctional. Class I and II CODH form heterotetramers containing nine iron–sulfur clusters (Fig. 1c). CODH of class III and IV are homodimeric, and contain three iron–sulfur clusters (Fig. 1b).

As exemplified by the photosynthetic bacterium *Rhodospirillum rubrum* (Rr), CO can be used as an alternative to light as a source of energy [16]. It is hypothesized that cytoplasmic oxidation of CO is coupled to the reduction of protons. The product dihydrogen would diffuse through the internal membrane and be oxidized by a periplasmic hydrogenase, leading to the formation of a proton motive force [3, 17, 18]. Some anaerobic sulfate reducing microorganisms couple the oxidation of CO to the reduction of sulfate [19–21] and some methanogenic archaea use CO as carbon/electron donor for methane formation [22, 23].

In cases where CODH is in complex with acetyl-CoA synthase (ACS), and is therefore called bifunctional, it reduces CO_2_ to CO which is channeled to ACS, whose active site (called A-cluster) is a bi-Ni center attached to an Fe–S cluster [24, 25]. This CODH/ACS complex is found in some anaerobic microorganisms, such as acetogenic and sulfate-reducing bacteria and methanogenic archaea, where CO_2_ is used as a source of carbon through the Wood–Ljungdahl pathway [26–29]. The major function of CODH in this metabolic pathway requires coordination of CO_2_ reduction at the C-cluster with CO channeling and reaction with a methyl group and CoA at the A-cluster active site of ACS.

This article reviews the mechanisms and accessory proteins/chaperones involved in the maturation of this unique C-cluster. There has been a general agreement that CODH maturation depends on the accessory protein CooC, but on the basis of the recent literature, it appears that the maturation of CODH may differ from one enzyme to another, and that in some cases, it is independent of any specific maturase.

## Maturation of the [Ni–4Fe–4S] C-cluster of CODH

**Fig. 2:**
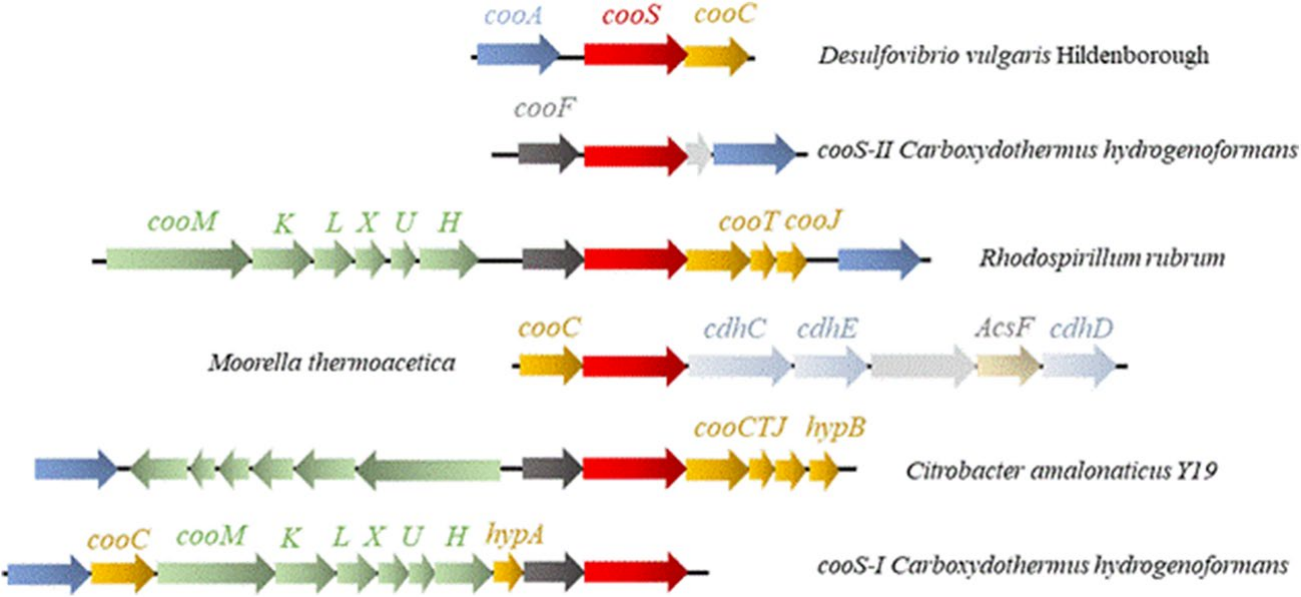
Operons encoding for CODH in *Desulfovibrio vulgaris Hildenborough* (Dv), *Carboxydothermus hydrogenoformans* Z-2901 (Ch), *Rhodospirillum rubrum* ATCC 11170 (Rr), *Moorella thermoacetica* and *Citrobacter amalonaticus* Y19 (CaY19). The operons coding for Ch CODH I and II are represented in this figure. Arrows in red represent the *cooS* genes encoding for CODH, in yellow genes encoding for maturation proteins, in blue genes encoding for the CO-dependent transcriptional activator cooA, in dark gray genes encoding for ferredoxin-like proteins, in green genes encoding for hydrogenase subunits, in gray-blue genes encoding for acetyl-CoA synthase subunits and in light gray genes encoding for hypothetical proteins of unknown functions

The diversity of the operons encoding for CODH (Fig. 2) suggests a variability in the maturation mechanisms. Some operons encoding for a CODH do not contain any maturase (e.g., CODH-II from Ch [27]), whereas many of them contain the maturation gene *cooC*. Other accessory genes can be present in the CODH operons in addition to *cooC*: for example, in *Rhodospirillum rubrum* (Rr) [16] and *Citrobacter amalonaticus* Y19 (CaY19) [30], the operons also contain the *cooJ* and *cooT* genes.

## Physiological importance of accessory proteins

The physiological functions of *cooC*, *cooT* and *cooJ*, when present in CODH operons, have mostly been studied in the photosynthetic bacterium *Rhodospirillum rubrum*. The group of Ludden has shown that, in the absence of light, the bacterium grows using CO as energy source. This growth depends on CODH: a Rr strain in which *cooS* has been inactivated grows normally under light but does not grow in the presence of CO in the dark [16]. Strains of Rr in which the genes coding for CooC, CooT or CooJ have been inactivated do not grow unless nickel is added to the growth medium [16]. The presence of CooC is particularly crucial, since only a large excess of nickel (650 µM) restores the growth of the *cooC*-inactivated strain. Therefore, these accessory proteins are important to ensure that CODH is fully Ni-loaded and active when nickel is scarce. However, their precise action and their interplay remain unknown. They may be directly involved in nickel delivery into the active site of CODH or act indirectly by being involved in intracellular nickel transport. Note that they are probably exclusively dedicated to CODH maturation: they are not involved in import of exogenous nickel [31] and inactivating these genes has no effect of the Ni–Fe hydrogenase activity [32, 33].

## Biochemical and structural characterization of the accessory proteins

### CooC

CooC is a 30 kDa ATPase which belongs to the MRP/MinD family of the SIMIBI class (Signal recognition GTPases, MinD superfamily and BioD superfamily) [34]. Rr CooC and Ch CooC-1 hydrolyze ATP at a slow rate in vitro (*k*_cat_ of 5 nmol min^−1^ for Ch CooC) [35, 36] *Rr* CooC also hydrolyzes GTP. The in vitro NTPase rates, although low, are in the same range as those of other Ni-metallochaperones which hydrolyze GTP (e.g., HypB and ureG, which are involved in the maturation of NiFe hydrogenase and urease, respectively) but hydrolysis might occur at a faster rate in the cellular context.

**Fig. 3:**
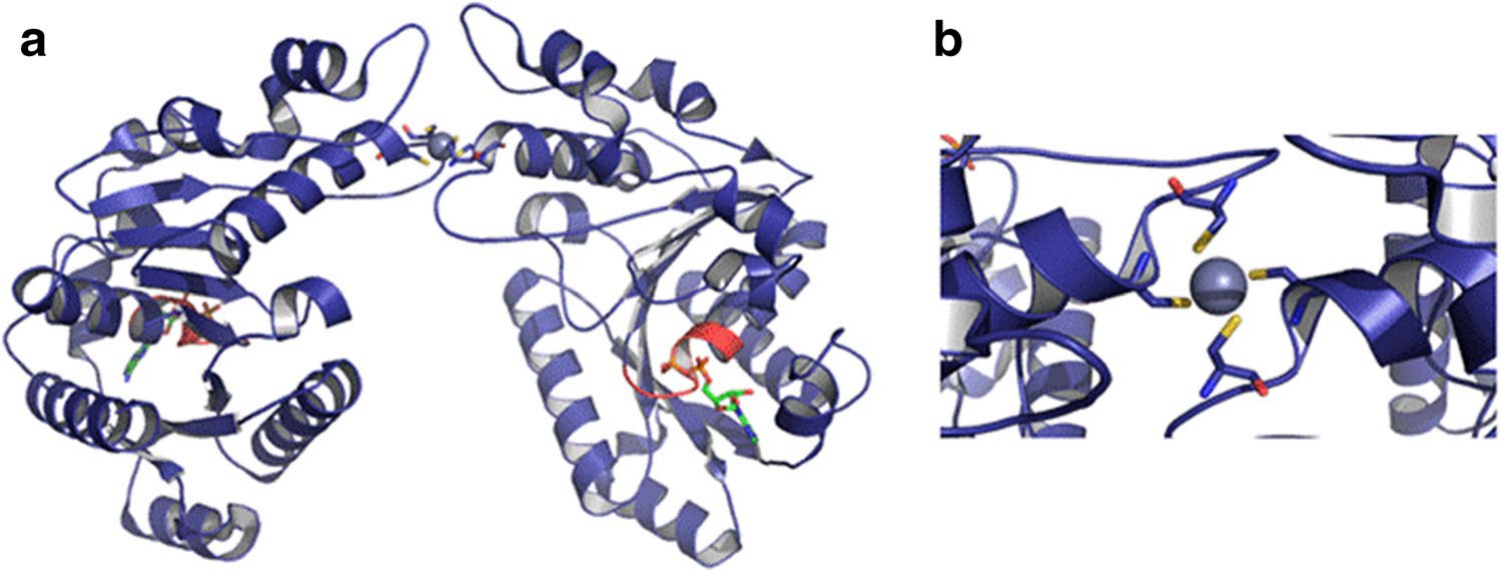
**a** Structure of CooC-1 from *Carboxydothermus hydrogenoformans*, PDB: 3KJI [40]. **b** An atom of zinc in gray is at the interface of the two monomers, bound by cysteines 112 and 114. ADP is bound to the deviant Walker A motif (GKGGVGKTT), shown in red

Ch CooC-1 is monomeric [35, 37] and forms a dimer in the presence of ADP and/or Zn^2+^ (Fig. 3a). According to the structure of *Ch* CooC-1 (Fig. 3), ADP binds to the deviant Walker A motif (GKGGVGKTT) in the P-loop (phosphate-binding loop). Zinc is bound covalently at the interface of two monomers by two strictly conserved cysteines of each monomer (Cys112 and Cys114) (Fig. 3b). The structure of Ch CooC-1 in the presence of nickel and/or ATP has not been determined. However, Jeoung et al. showed that CooC-1 binds one nickel per dimer with a *K*_d_ of 0.4 µM [35] and that Zn and Ni compete for the same tetracoordination site at the interface of the dimer [37]. Whether all CooC proteins bind nickel is, however, unclear: the protein from Rr does not tightly bind nickel (Ni content lower than 0.1 Ni ion per dimer) under the conditions used in the study of Jeon et al. [36].

The available structures of Ch CooC-1 show that ADP binding induces conformational changes: the distance between the metal (Zn) and the thiolate group of cysteine 114 becomes shorter (from 2.5 to 2.2 Å) and a flexible loop, the CAP loop, deviates from the metal binding site, making it more open to metals [37]. Thus, the presence of ADP seems to favor nickel binding. The group of Dobbek has constructed a structural model of ATP-bound CooC, based on the structure of an ATPase of the same family involved in chromosome segregation [38]. They proposed that ATP binding increases the distances between the cysteines of the metal binding site, which induces the release of nickel [37].

### CooT

Timm et al. have recently resolved the crystal structure of CooT from Rr [39]. It is a small protein of 7.1 kDa, which dimerizes and binds one nickel per dimer with a *K*_d_ of 9 nM. The crystal structure of CooT shows that the protein is composed of seven β-sheets. Site-directed mutagenesis and circular dichroism experiments showed that the position 2 cysteine is involved in nickel binding. The authors hypothesized that Cys2 from each monomer coordinates nickel. However, no crystal structure of Ni-bound CooT could be obtained.

Using a bioinformatic search on genomes, Timm et al. have identified 111 proteins homologous to CooT. Among the CooT-containing proteomes, 85 also contain CODH [39], showing that CooT-dependent maturation of CODH is probably more widespread than was previously expected.

### CooJ

Rr CooJ is a small protein of 12.6 kDa which has a histidine-rich domain (16 histidines in the C-terminal domain). Biochemical studies revealed that CooJ binds four nickel per monomer with a dissociation constant of 4.3 µM [40], but its precise role in Ni cluster assembly is unknown.

## CooC-dependent maturation

Let us first focus on the cases in which CODH are encoded in a operon also containing *cooC*.

### Production of carbon monoxide dehydrogenases in the absence of CooC

The heterologous production of Ch CODH-I in *E. coli,* in the absence of CooC-1 leads to the formation of a an enzyme that contains three times less nickel, and is three times less active than when it is expressed in the presence of CooC [41]. Similarly, the CODH from *Desulfovibrio vulgaris* Hildenborough (Dv) produced heterologously in *D. fructosovorans* in the absence of CooC contains less than 0.5 nickel per dimer, compared to 0.8–1.8 Ni/dimer when it is co-produced with CooC [5]. Dv CODH is inactive when produced without CooC and cannot be activated with exogenous Ni. In both cases, it therefore appears that CooC is important for nickel insertion into the active site, even if the effect is more pronounced for the Dv enzyme.

Jeon et al. showed that the hydrolysis of ATP by CooC in Rr is necessary for nickel delivery and the production of a fully matured CODH [36]. Indeed, a mutated Rr strain in which CooC has no ATPase activity (K13Q CooC) produces a nickel-deficient and almost inactive CODH. Addition of nickel to the culture medium does not compensate for the deficiency of CooC [36]. As mentioned before, the inactivation of *cooC* prevents the CODH-dependent growth unless a large concentration of nickel is added in the medium [16]. This suggests that growth can be sustained even if the CODH is only partly Ni-loaded.

The UV visible spectra of Dv CODH obtained in the absence or in the presence of CooC are similar [5]. The absence of CooC does not affect the iron content of CODH, which suggests that CooC is not involved in the biosynthesis of the iron–sulfur clusters of CODH (including that of the active site). These iron–sulfur clusters are probably produced through generic iron–sulfur cluster assembly machineries, while CooC is specifically devoted to the delivery of nickel.

The heterologous production of the complex carbon monoxide dehydrogenase/acetyl-coA synthase from *Clostridium carboxidivorans* (Cc) in *Clostridium acetobutylicum* induces the production of CO by the host organism. In the absence of added nickel, the presence of Cc CooC enhances CO production. On the contrary, the CO production does not depend on Cc CooC when the medium is supplemented in nickel (50–100 µM). This study supports the idea that CooC facilitates nickel insertion into the active site of CODH when nickel is at trace levels [42].

CooC is not specific to the maturation of monofunctional CODH, the heterologous maturation of the CODH from *Moorella thermoacetica,* which is in complex with acetyl-CoA synthase, also seems to depend on the presence of this accessory protein, based on the results of experiments where the bifunctional enzyme was produced in *E. coli* [6].

### Proposed mechanisms of CooC-driven nickel insertion into the active site

Two different mechanisms for the maturation of the active site of CODH in which CooC is essential are proposed in the literature. In a first mechanism, CooC binds nickel and inserts it into the active site of CODH in reaction that is coupled to ATP hydrolysis [37]. In a second mechanism, CooC acts as a chaperone that induces a conformational change of the active site of CODH by hydrolyzing ATP, and then the folded active site of CODH spontaneously binds Ni [36].

We argued that the latter mechanism is more likely to be operational, since the Dv CODH produced in the absence of its maturase CooC, contains hardly any nickel, is inactive and cannot be activated in vitro (with nickel under reducing conditions) contrary to the enzyme that has been co-produced with CooC [5]. Similar observations were reported for Rr CODH: a strain producing a deficient CooC produces a nickel-depleted CODH which can only be partially activated with nickel under reducing conditions (to approximately 15% of the wild-type CODH activity); these CODH cannot be fully activated in vitro, which suggests that the active site has not the right conformation to bind nickel [5, 36]. As a matter of fact, conversely, when WT Rr is grown in a Ni-depleted medium, the purified CODH does not contain Ni but activates upon incubation with NiCl_2_ under reducing conditions. This shows that the presence of CooC influences the ability of CODH to bind Ni. CooC is the Ni donor in the case of Ch CooC-1, but it may be that this function is performed by accessory proteins (CooJ and CooT) in the case of organisms such as Rr, whose CooC does not bind Ni. In the case of Dv and Rr, free Ni can be delivered directly in the active site of CODH, at least in vitro, provided the enzyme has been co-produced with CooC.

**Fig. 4:**
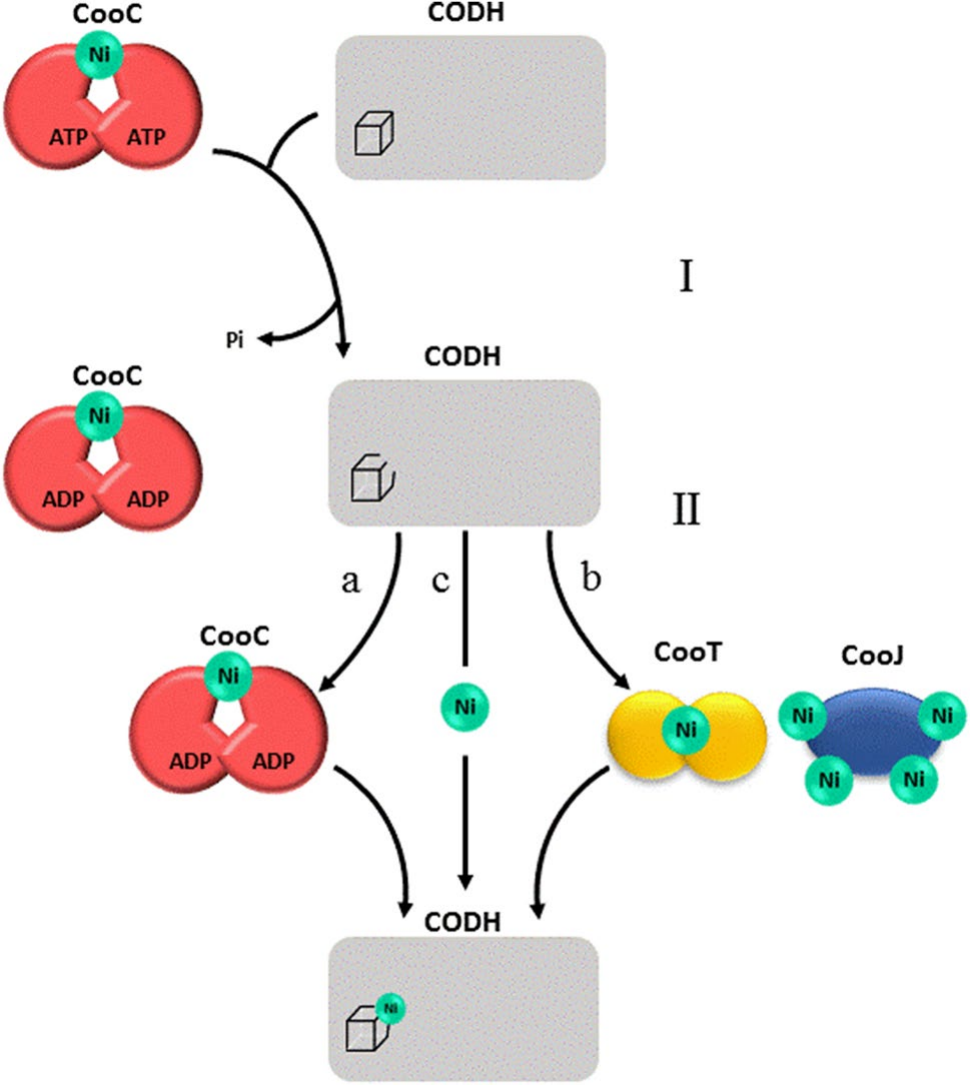
Proposed mechanisms of CooC-dependent CODH maturation. Step I: CooC derives energy from the hydrolysis of ATP to induce a conformational change of the active site. Step II: the active site is in a favorable folding to receive nickel. The active site of CODH may acquire nickel from **a** nickel-loaded CooC, **b** other nickel-loaded accessory proteins (CooJ and CooT) or **c** free nickel available in the environment

We depict the current hypothesis in Fig. 4. In the first step, CooC acts as a chaperone which properly folds the active site in a conformation that makes it ready to bind Ni, at the cost of ATP hydrolysis. In a second step, nickel binds to the active site according to three possible routes depending on the organism: (a) it can be delivered by Ni-loaded CooC itself if its affinity for Ni is high (Ch CODH-I), (b) if CooC has low affinity for Ni (Rr CODH), CooT and CooJ assist it for the delivery of nickel into the folded active site, and (c) free Ni can possibly be inserted without the need for another protein (Dv and Rr CODH).

Like what we propose for some CooC proteins, maturases with double function have already been described. NifH, involved in the maturation of nitrogenase, for instance, is an ATPase that belongs to the MinD family. It binds a [4Fe–4S] cluster at the interface of the two monomers [43]. First, hydrolysis of ATP by NifH induces a conformational change which results in electron transfer from the [4Fe–4S] cluster to the nitrogenase. Then NifH is involved in the biosynthesis of the molybdenum iron cofactor, by delivering homocitrate and molybdenum [44, 45]. More in-depth studies and comparisons of CooC from different sources are required to support our hypothesis.

### Maturation without any specific accessory protein

Surprisingly, whereas the maturation of some CODH is strictly dependent on the presence of one or several maturases, other CODH can be fully matured in the absence of any specific accessory protein.

In organisms that express several CODH, operons coding for certain CODH do not contain genes encoding for accessory proteins, which suggests that these CODH may either depend on accessory proteins encoded by other CODH operons (for example, the Ch CODH-II and -IV operons do not contain a *cooC* copy but the CODH-I and III operons do) or that their maturation is not assisted by any accessory protein [27].

The heterologous production in *E. coli* of Ch CODH-II or -IV, for instance, leads to the formation of nickel-loaded, mature and active CODH [8, 46, 47]. It is important to note that in these studies *E. coli* is grown in the presence of high concentrations of nickel (more than 0.5 mM) to favor nickel insertion into CODH. It may be that the high concentration of nickel compensates for the absence of accessory proteins. This is probably the case for the CODH from *Citrobacter amalonaticus* Y19 (CaY19). The *cooS* gene from CaY19 precedes four genes encoding for accessory proteins: CooC, CooT, CooJ and HypB (Fig. 2) [48]. Some CODH activity could be measured in crude extracts of an *E. coli* strain in which the *Ca*Y19 CODH was produced in the absence of the accessory proteins, suggesting that the latter are not strictly necessary for Ni delivery to the active site. However, the actual metal content of the heterologously produced CODH was not determined so that it is unknown whether the enzyme was fully Ni-loaded. Surprisingly, among the proteins encoded in the *coo* operon, only CooF is essential for the formation of an active *Ca*Y19 CODH. CooF is a small iron–sulfur-containing protein (22 kDa for CooF of Rr) which probably shuttles electrons from CODH to the CO-induced Ni–Fe hydrogenase Coo [17, 49]. Its potential function in *Ca*Y19 CODH maturation remains unknown.

Regarding heterologous expression, another hypothesis is that accessory proteins of other Ni-containing enzymes, such as Ni–Fe hydrogenases, from the heterologous host may be involved in the maturation of CODH [6, 48]. In *Helicobacter pylori*, it is well known that Ni–Fe hydrogenase accessory proteins can maturate another nickel enzyme, urease. Deletions of *hypA* and *hypB* cause a decrease in urease activity (40- and 200-fold, respectively) compared to the wild-type strain [50]. Site-directed mutagenesis studies showed that the GTPase activity of HypA and the nickel-binding site of HypB are involved in the maturation of the urease of *H. pylori* [51, 52]. On the contrary, accessory proteins of urease from *H. pylori* cannot maturate Ni–Fe hydrogenase [53, 54]. Similar conclusions have been drawn regarding the metallochaperones CooC, CooT and CooJ from Rr which are not involved in the maturation of the CO-induced Ni–Fe hydrogenase (referred to as Coo hydrogenase) [32]. Although the cross-talk between maturation machineries of different Ni-containing enzymes is established, the involvement of hydrogenase accessory proteins, such as HypA and HypB, in the Ni-acquisition of CODH remains to be demonstrated.

Last, we note that there are also examples of genomes that contain a CODH but no *cooC* gene, which definitely establishes that CODH maturation can be CooC-independent. It is so in *C. acetobutylicum,* whose gene CA_C0116 is annotated as a CODH and codes for a protein that has all amino acids known to be essential for CODH function (this is unlike the CA_C2498 gene which is annotated as a CODH but lacks one cysteine residue of the C-cluster and probably codes for a hybrid cluster protein). Preliminary results in our group show that indeed, expression of CA_C0116 in the absence of any CooC leads to a functional CODH.

## Concluding remarks

Although small in numbers, Ni-containing enzymes exhibit a remarkable diversity in terms of active site architecture and catalytic properties. They are often involved in crucial metabolic pathways, catalyzing reactions related to carbon fixation, energy conversion or pathogenicity in many microorganisms. The structure of the active site of some of these enzymes is so elaborated that accessory proteins are necessary to bind the Ni ion and insert it. This is the case for NiFe hydrogenase, urease, lactate racemase and methyl-coM reductase [55–58]. As also noted by Zeer-Wanklyn and Zamble [59], the common point of these maturation processes is that they depend on an NTP hydrolysis, which supplies the required energy. Focusing on CODH, CooC (sometimes assisted by CooJ and CooT), is an ATPase involved in Ni insertion in the active site but the mechanism involved remains undetermined. Examination of the literature reveals that not all CODH are CooC-dependent, which is surprising considering that the active site of all Ni-containing CODH is believed to be the same. It is not clear in these cases whether CooC is substituted by the Ni insertion machinery of other Ni-enzymes or if Ni spontaneously binds the active site. Understanding how CooC works and how and why some CODH do not depend on this accessory protein is certainly part of the most fascinating questions in the field.
