# Correction to: Maturation of the [Ni–4Fe–4S] active site of carbon monoxide dehydrogenases

**DOI:** 10.1007/s00775-018-1565-5

**Published:** 2018-05-29

**Authors:** Mériem Merrouch, Martino Benvenuti, Marco Lorenzi, Christophe Léger, Vincent Fourmond, Sébastien Dementin

**Affiliations:** Aix-Marseille Université, CNRS, BIP UMR 7281, Institut de Microbiologie de la Méditerranée, 31 Chemin J. Aiguier, 13402 Marseille Cedex 20, France

## Correction to: JBIC Journal of Biological Inorganic Chemistry 10.1007/s00775-018-1541-0

The article “Maturation of the [Ni–4Fe–4S] active site of carbon monoxide dehydrogenases”, written by Mériem Merrouch, Martino Benvenuti, Marco Lorenzi, Christophe Léger, Vincent Fourmond, Sébastien Dementin was originally published electronically on the publisher’s internet portal (currently SpringerLink) without open access.

The copyright of the article changed on 18, May to © The Author(s) 2018 and the article is forthwith distributed under the terms of the Creative Commons Attribution 4.0 International License (http://creativecommons.org/licenses/by/4.0/), which permits use, duplication, adaptation, distribution and reproduction in any medium or format, as long as you give appropriate credit to the original author(s) and the source, provide a link to the Creative Commons license and indicate if changes were made.

The original article has been corrected.

